# T-type calcium channel antagonists, mibefradil and NNC-55-0396 inhibit cell proliferation and induce cell apoptosis in leukemia cell lines

**DOI:** 10.1186/s13046-015-0171-4

**Published:** 2015-05-21

**Authors:** Weifeng Huang, Chunjing Lu, Yong Wu, Shou Ouyang, Yuanzhong Chen

**Affiliations:** Fujian Institute of Hematology, Fujian Medical University Union Hospital, Fuzhou, 350004 People’s Republic of China; Department of Blood Transfusion, Maternal and Child Health Hospital of Xiamen, Xiamen, 361003 People’s Republic of China; Xiamen Medical Research Institute, Xiamen, 361008 People’s Republic of China

**Keywords:** T-type calcium channels, Mibefradil, NNC-55-0396, Leukemia, Proliferation, Apoptosis

## Abstract

**Background:**

T-type Ca^2+^ channels are often aberrantly expressed in different human cancers and participate in the regulation of cell cycle progression, proliferation and death. Methods: RT-PCR, Q-PCR, western blotting and whole-cell patch-clamp recording were employed to assess the expression of T-type Ca^2+^ channels in leukemia cell lines. The function of T-type Ca^2+^ channels in leukemia cell growth and the possible mechanism of the effect of T-type Ca^2+^ channel antagonists on cell proliferation and apoptosis were examined in T-lymphoma cell lines.

**Results:**

We show that leukemia cell lines exhibited reduced cell growth when treated with T-type Ca^2+^ channel inhibitors, mibefradil and NNC-55-0396 in a concentration-dependent manner. Mechanistically, these inhibitors played a dual role on cell viability: (i) blunting proliferation, through a halt in the progression to the G1-S phase; and (ii) promoting cell apoptosis, partially dependent on the endoplasmic reticulum Ca^2+^ release. In addition, we observed a reduced phosphorylation of ERK1/2 in MOLT-4 cells in response to mibefradil and NNC-55-0396 treatment.

**Conclusions:**

These results indicate that mibefradil and NNC-55-0396 regulate proliferation and apoptosis in T-type Ca^2+^ channel expressing leukemia cell lines and suggest a potential therapeutic target for leukemia.

**Electronic supplementary material:**

The online version of this article (doi:10.1186/s13046-015-0171-4) contains supplementary material, which is available to authorized users.

## Introduction

Intracellular Ca^2+^ is a crucial secondary messenger that regulates many cellular processes, such as cell cycle progression, proliferation and apoptosis [1–3]. Intracellular Ca^2+^ levels are regulated by several mechanisms including plasma membrane ion channels (e.g., Voltage-gated and ligand-gated Ca^2+^ channels), ion exchangers and “pumps”, as well as the release from the intracellular Ca^2+^ stores [3]. Orchestration of cytoplasm Ca^2+^ as evidenced by pulses, or oscillations, is crucial for cell cycle progression and therefore proliferation [4], otherwise, excessive Ca^2+^ or loss of control in Ca^2+^ signaling can lead to cell death [5]. In normal epithelial cells, free Ca^2+^ concentration is essential for cells to enter and accomplish the S phase and the M phase of the cell cycle. Cancer cells are able to pass these phases with much lower extracellular Ca^2+^ levels than normal cells [6], indicating that they developed a more efficient mechanism to facilitate Ca^2+^ influx.

Among the routes for Ca^2+^ influx, T-type Ca^2+^ channel expression and relationship to proliferation and apoptosis have been demonstrated in many cancer types, including leukemic [7], ovarian [8, 9], glioma [10, 11], breast [12], esophageal [13], hepatoma [14], melanoma [15], and colon cancers [16]. Moreover, increased expression of T-type Ca^2+^ channels can be detected in tumor samples collected from patients. In addition, these reports also show that pharmacological inhibition by small molecule antagonists or RNAi-mediated downregulation of T-type Ca^2+^ channels leads to inhibition of cancer cell proliferation and inducing cancer cell apoptosis. Therefore, T-type Ca^2+^ channels pose an attractive potential target for cancer therapy. T-type Ca^2+^ channels have unique electrophysiological characteristics: low voltage-activated Ca^2+^ current, fast (transient) inactivation, slow deactivation and low unitary conductance [17]. To date, the existence of three different T-type Ca^2+^ channel subunits, the α1G (Ca_v_3.1), α1H (Ca_v_3.2) and α1I (Ca_v_3.3) has been revealed [17]. At low voltages, T-type Ca^2+^ channels are known to mediate a “window current” [18], i.e. a sustained inward Ca^2+^ current carried by the portion of channels that are not completely inactivated. Hence, T-type Ca^2+^ channels are well suited to regulate Ca^2+^ oscillations under non-stimulated or resting membrane conditions. This regulation of Ca^2+^ homeostasis allows T-type Ca^2+^ channels to control cell proliferation and apoptosis, or death. There are increasing data suggest that the expression of T-type Ca^2+^ channels is cell cycle-dependent [19–22].

Mibefradil is a potent inhibitor of T-type Ca^2+^ currents with 10 to 20 times higher selectivity for T-type over L-type Ca^2+^ channels [23]. NNC-55-0396, is a structural analog of mibefradil with a higher selectivity for T-type Ca^2+^ channels, which exerts no effect against high voltage Ca^2+^ channels at 100 μM, but inhibits T-type Ca^2+^ channels in HEK293 cells with a potency comparable to that of mibefradil (IC_50_ values of 6.8 versus 10.1 μM) [24]. A growing number of reports showed that mibefradil and NNC-55-0396 could prevent human cancer cell proliferation and induce cancer cell apoptosis as a result of its ability to inhibit the function of T-type Ca^2+^ channels [10–16, 23, 24]. Additionally, mibefradil was FDA-approved for the treatment of ovarian (2007), pancreas (2008), and glioblastoma multiforme (2009) tumors. At present, however, the detailed biological mechanism (s) underlying the anticancer activity of these channel antagonists has not been explored.

In this study, we examined the function of T-type Ca^2+^ channels in leukemic cell lines. We showed that inhibition of T-type Ca^2+^ channels with antagonists, mibefradil and NNC-55-0396, led to a decrease in proliferation, and an increase in apoptosis of leukemia cells in vitro, which was preceded by disrupting endoplasmic reticulum (ER) Ca^2+^ homeostasis. We also demonstrated down-regulating ERK signaling in MOLT-4 cells following the application of T-type Ca^2+^ channel antagonists. Since human normal blood cells do not express T-type Ca^2+^ channels, our results suggest that T-type Ca^2+^ channel inhibitors may be useful in the treatment of acute lymphocytic leukemia (ALL).

## Materials and methods

### Cell culture

Human leukemic cell lines MOLT-4, Jurkat, Ball, HL-60, NB4, HEL, K-562, and U937 were purchased from the American Type Culture Collection (ATCC; Rockville, MD, USA) and were cultured in RPMI 1640 medium containing 10 % heat-inactivated fetal bovine serum (Gibco by Life Technologies, Carlsbad, CA, USA), 1 % pen/strep (MP Biomedicals, Solon, OH, USA) and 2 mM L-glutamine at 37 °C in 95 % air/5 % CO_2_ with 95 % humidity.

### Isolation of human peripheral blood mononuclear cells (PBMCs)

Whole blood (5–10 ml) was collected from healthy human male and female donors (*n* = 8 each), according to The Code of Ethics of the World Medical Association. Mononuclear cells were isolated with human lymphocyte separation medium (Tbdscience, Tianjin, China) according to manufacturer’s instructions. Briefly, PBMCs were separated by centrifugation at 900 × g for 30 min at 18–20 °C over a Ficoll-Paque PLUS gradient. The resulting PBMC layer was washed twice with nuclease-free 0.9 % NaCl solution and prepared for RNA isolation.

### Reverse transcriptase-polymerase chain reaction (RT-PCR)

Total cellular RNA was isolated from exponentially growing cells and human PBMCs using RNAsimple Total RNA Kit (TIANGEN Biotech, Beijing, China). Messenger RNA was reverse-transcribed (RT) to cDNA using oligo(dT)_15_ primers and GoScript reverse transcriptase (Promega, Madison, WI, USA). The cDNA product was used as a template for subsequent PCR amplifications for α1G, α1H, and α1I subunit, using sequence-specific primers. Primer sequences, product sizes and PCR conditions are summarized in Table 1. PCR analysis was repeated at least three times with the same samples to confirm reproducibility of the results.

**Table 1:** Oligonucleotides used to amplify transcripts of T-type Ca^2+^ channel α1 subunits and GAPDH

Target	Sequence	Product size(bp)	Temp.
α1G	F: 5′-TGCTCTGCTTCTTCGTCTTCTT -3′	152	60.0 °C
	R: 5′-CTCATCCTCGTTCTCTGTCTGGT-3′		
α1H	F: 5′-TTGGGTTCCGTCGGTTCT-3′	193	56.5 °C
	R: 5′-ATGCCCGTAGCCATCTTCA-3′		
α1I	F: 5′-ATCGGTTATGCTTGGATTGTCA-3′	203	54.0 °C
	R: 5′-TGCTCCCGTTGCTTGGTCTC-3′		
GAPDH	F: 5′-AGAAGGCTGGGGCTCATTTG-3′	258	57.5 °C
	R: 5′-AGGGGCCATCCACAGTCTTC-3′		

### Quantitative PCR

Total RNA 1 μg was used to generate cDNA with GoScript reverse transcriptase as above. A 1-μl aliquot of each synthesized cDNA was analyzed by Quantitative Real-Time PCR (CFX96 Real-Time System, Bio-Rad, Singapore) using SYBR Green PCR Master Mix (Takara, Dalian, China) according to manufacturer’s protocols and message level was determined using the △C_t_ method. Samples were assayed in triplicate for each gene, and the mean expression was used during subsequent analysis. Q-RT-PCR was carried out under the following reaction conditions: stage 1, 95 °C for 30 s (Rep 1); stage 2, 95 °C for 5 s then 60 °C for 1 min (Reps 40).

### Western blot analysis

Western blotting was performed as described previously [25, 26]. Immunoblots were developed with a goat anti-rabbit horseradish peroxidase-conjugated secondary antibody (1:10,000; Santa Cruz Biotechnology, Santa Cruz, CA, USA) incubated for 1 h at room temperature. Immunoblots were visualized with the ECL immunodetection system (Advansta, Menlo Park, CA, USA). The following primary antibodies were used: anti-Cav3.1(1:200 dilution, rabbit polyclonal, Alomone Labs, Israel), anti-Cav3.2 (1:200 dilution, rabbit polyclonal, Alomone Labs) and anti- Cav3.3 (1:200 dilution, rabbit polyclonal, Alomone Labs), anti-ERK1/2 and anti-pERK1/2 (1:1000 dilution, rabbit polyclonal; Cell Signaling, Beverly, MA, USA), and anti-GAPDH (1:1000 dilution, rabbit polyclonal, Goodhere, Hangzhou, China).

### Whole-cell patch-clamp recording

Whole-cell voltage-clamp recordings were performed by following the procedures as described in our previous studies [27]. For T-current recordings, the cells were superfused with bath solution containing (in mmol/L): 10 HEPES, 110 TEA-Cl, 10 CsCl, 20 BaCl_2_, 10 glucose, pH 7.4 adjusted with TEA-OH. The resistance of pipettes ranged 3–5 MΩ when filled with internal solution containing (in mmol/L): 10 HEPES, 120 CsCl, 1 MgCl_2_, 10 TEA-Cl, 10 EGTA, 5 Na_2_ATP, 1.2 Creatine phosphase, pH 7.2 adjusted with CsOH. Liquid junction potential was not compensated. Following whole cell access, the cells were held at- 80 mV with test pulses ranging from −60 mV to +60 mV with 10 mV increments.

### Cell growth assay

To determine cell survival and proliferation, cell growth was quantified using the CellTiter 96 AQ One Solution Cell Proliferation Assay Kit (Promega, Madison, WI, USA). Cells were plated in 96-well culture plates at a density of 1–2 × 10^4^ cells/well in 100 μL of cell culture media. Cells were treated with different concentrations of mibefradil or NNC-55-0396 (Sigma-Aldrich, St. Louis, MO, USA). After drug exposure, 20 μL of CellTiter 96 AQ One Solution Reagent was added to each well and allowed to incubate for 2 h at 37 °C. The quantity of formazan product formed, which is directly proportional to the number of viable cells, was measured on a Multi-Mode Microplate Reader (MD SpectraMax M3, CA, USA) at 490 nm wavelength using a reference filter at 650 nm wavelength. Viability assays were performed at least three times in independent experiments, using triplicate measurements in each.

### RNAi against α1G and α1H

The target sequence against human both Ca_v_3.1 and Ca_v_3.2 T-type Ca^2+^ channels was designed according to a previous report [12]. RNAi oligonucleotides (Ca_v_3.1/3.2, 5′- GCCATCTTCCAGGTCATCACA -3′; negative control scramble sequence, 5′-TTCTCCGAACGTGTCACGT-3′) were synthesized by Integrated DNA Technologies and cloned into the lentiviral small interference RNA (siRNA) vector GV115 (GeneChem, Shanghai, China). Transduction of shRNA into the MOLT-4 cells was achieved by a lentiviral infection method. The positive transfected cells were sorted using the flow cell sorter and subjected to the CellTiter 96 AQ One Solution Cell Proliferation Assay. Q-RT-PCR was used to verify that shRNA decreased T-type Ca^2+^ channel genes expression.

### Flow cytometer cell cycle analysis

Analysis of cell cycle distribution was determined by propidium iodide (PI) staining and flow cytometry according to manufacturer’s instructions (Keygen Biotech, Nanjing, China). Briefly, following treatment, approximately 1 × 10^6^ cells were fixed in 70 % ethanol for 2 h on ice. The cell pellets were washed with PBS and incubated with 100 μL RNase A solution for 30 min at 37 °C. PI (400 μL) was then added and allowed to incubate for an additional 30 min at 4 °C in dark. DNA content was measured by exciting PI at 488 nm and measuring the emission at 620 nm, using a flow cytometer (BD Accuri C6, Ann Arbor, MI, USA). Data analysis was carried out using FlowJo software. Each experiment is representative of at least three independent experiments.

### Apoptosis assay

Apoptosis of ALL cells was detected using an annexin V apoptosis assay, followed by flow cytometry analysis. In brief, cells were harvested following treatment, washed in PBS, and subjected to Annexin V/PI staining according to the manufacturer’s protocol (Keygen Biotech, Nanjing, China). The percentage of apoptotic cells was evaluated using flow cytometer (BD Accuri C6).

### Measurement of Intracellular Ca^2+^ Levels

Briefly, cells were loaded with 1 μM Fluo-4/AM (Invitrogen) for 60 min at 37 °C in 1640 medium, washed 3 times with PBS and resuspended in 1640 or calcium-free medium. The loaded cells were measured by flow cytometry in a FACScan (BD Accuri C6) at an excitation wavelength of 488 nm and an emission wavelength of 520 nm as described below.

### Determination of mitochondrial membrane potential

Mitochondrial membrane potential, ψ_m_, was assessed with 5, 5′, 6, 6′-tetrachloro-1, 1′, 3, 3′-tetraethylbenzimidazolylcarbocyanine iodide fluorescent probe (JC-1) (Beyotime, Nantong, China). The treated and control cells were harvested and incubated with JC-1 for 20 min at 37 °C in the dark. The cells were washed and resuspended in 100 μL of cold PBS and then analyzed with flow cytometer (BD Accuri C6).

### Statistical analysis

Plots were produced using Origin 7.0 (Microcal Software, Inc., Northampton, MA). Results were compared using unpaired t-tests (for comparing two groups) or one factor ANOVA analysis followed, where appropriate, by Student-Newman-Keuls (for multiple comparisons) post-test. A p-value of less than 0.05 indicated statistically significant differences between observed effects. The results are expressed as mean ± SEM.

## Results

### The expression of T-type Ca^2+^ channels in human leukemia cell lines and PBMCs

**Fig. 1:**
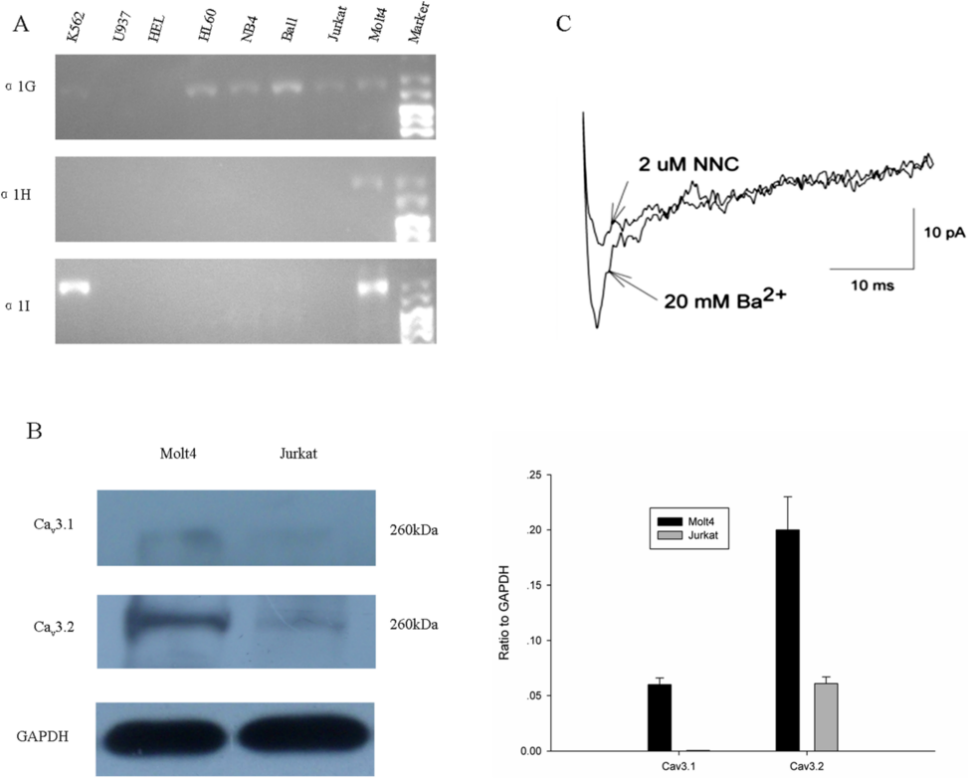
Expression of T-type Ca^2+^ channels in human leukemic cell lines. **a** RT-PCR expression analysis of T-type Ca^2+^ channel α1-subunits in human leukemic cell lines. α1G:35 cycles, α1H: 35 cycles, α1I: 37 cycles. **b** The protein expression levels of T-type Ca^2+^ channel α1-subunits (Ca_v_3.1 and Ca_v_3.2) in MOLT-4 and Jurkat cells were determined by Western blot analysis. Right panel showed relative protein expression levels of Ca_v_3.1 and Ca_v_3.2 compared to GAPDH. **c** T-type Ca^2+^ currents recorded in a MOLT-4 cell before and after application of 2 μM NNC-55-0396 in 20 mM Ba^2+^-containing bathing solution. The holding potential was −80 mV and the test potential was 0 mV. NNC, trace recorded 5 min after 2 μM NNC-55-0396 was perfused into the bath

We first examined the expression of T-type Ca^2+^ channels in human leukemia cell lines (MOLT-4, Jurkat, Ball, HL-60, NB4, HEL, K-562 and U937) using standard reverse transcriptase PCR (RT-PCR). As shown in Fig. 1a, these leukemia cell lines examined expressed mRNA for the T-type α1-subunit except HEL and U937 cells: either α1G alone (e.g., Jurkat, Ball, HL-60, and NB4), α1G and α1I (K-562), or all three T-type α1 subunits (MOLT-4). We additionally determined the quantitative expression of these channels by qPCR in human leukemia cell lines and human PBMCs. As shown in Table 2, except α1H in MOLT-4 cells showed high level, the expression of T-type α1 subunits in other cells were very weak or negative, while human PBMCs didn’t express T-type Ca^2+^ channels. We also examined the expression of T-type Ca^2+^ channels in MOLT-4 and Jurkat T cells employing western blot analysis. Both Ca_v_3.1 (very weak) and Ca_v_3.2 subunits were stained in a size of ~260 kD from the cellular extract of MOLT-4 cells, whereas only Ca_v_3.2 subunit (very weak) was detected in Jurkat cells (Fig. 1b).

**Table 2:** Q-RT-PCR detected T-type Ca^2+^ channel α1 subunits expression on human leukemic cell lines and PBMCs (^Δ^Ct)

Target	MOLT-4	Jurkat	Ball	NB4	HL60	HEL	U937	K562	PBMCs
α1G	14.81 ± 0.57	14.37 ± 0.25	15.21 ± 0.27	18.12 ± 0.34	17.52 ± 0.31	NA, Ct > 40	NA, Ct > 40	NA, Ct > 40	NA, Ct > 40
α1H	10.69 ± 0.43	12.60 ± 0.39	NA, Ct > 40	NA, Ct > 40	NA, Ct > 40	NA, Ct > 40	NA, Ct > 40	NA, Ct > 40	NA, Ct > 40
α1I	17.55 ± 0.66	NA, Ct > 40	NA, Ct > 40	NA, Ct > 40	NA, Ct > 40	NA, Ct > 40	NA, Ct > 40	17.21 ± 0.36	NA, Ct > 40

To evaluate the functional expression of T-type Ca^2+^ channels in MOLT-4 T cells, whole-cell patch-clamp recordings were performed to record the T-type Ca^2+^ current. Using Ba^2+^ as a charge carrier, the current activated at −30 mV, with peak current at 0 mV, and displayed rapid activation and inactivation kinetics (Additional file 1: Figure S1). The amplitude of T-current in MOLT-4 cells varied between 10 and 20 pA (*n* = 8), and the mean T-current density was 0.69 ± 0.15 pA/pF. Figure 1c shows that a ~15 pA T-type Ca^2+^ current was elicited by a depolarizing pulse at 0 mV when held at −80 mV (control). After perfusion of 2 μM of NNC-55-0396, the T-type Ca^2+^ current was inhibited by ~70 %. In addition, MOLT-4 cells showed a mean resting potential of −30.5 ± 1.8 mV (*n* = 12) and membrane capacitance of 14.5 ± 0.7 pF (*n* = 15). Treatment of T lymphocytes with mibefradil, a selective inhibitor against T-type Ca^2+^ channels, blocked Ca^2+^ influx (Additional file 2: Figure S2). These findings indicate that T-type Ca^2+^ channels play a significant role in the Ca^2+^ influx pathways of human leukemia T cell line.

**Fig. 2:**
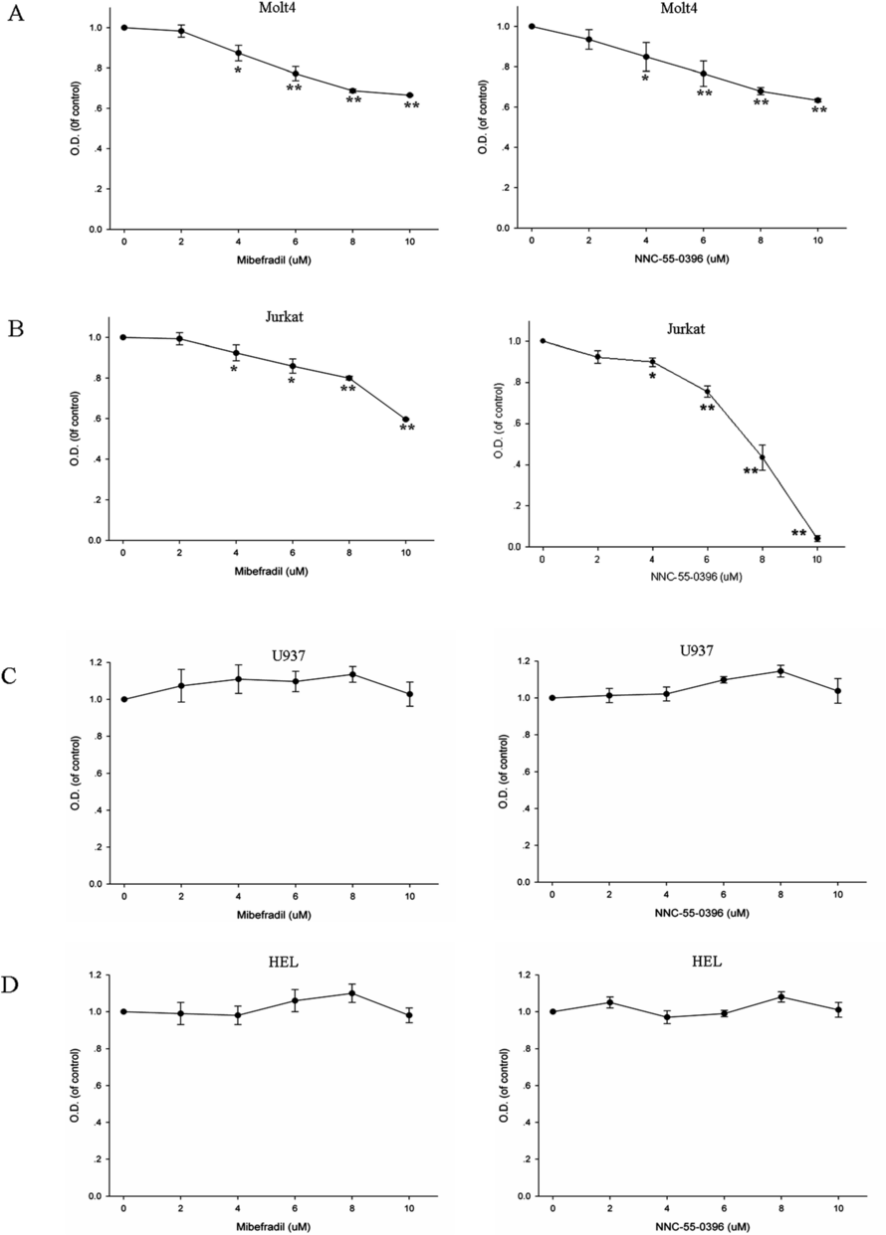
Effect of T-type Ca^2+^ channel blockers, mibefradil and NNC-55-0396 on cell growth. MOLT-4 (**a**), Jurkat (**b**), U937 (**c**) and HEL (**d**) cells were cultured in the present of mibefradil or NNC-55-0396 (2–10 μM) for 48 h. All data points represent an average of three to five experiments (± SEM). *P < 0.05 versus control, **P < 0.01 versus control

### T-type Ca^2+^ channels blockers reduced the viability of human ALL cells

Since T-type Ca^2+^ channels have been previously shown to be involved with cell proliferation, we wanted to examine a putative effect of selective T-type Ca^2+^ channel antagonists, mibefradil and NNC-55-0396 on the viability of ALL cells. As shown in Fig. 2a and b, the cell viability of both MOLT-4 and Jurkat was suppressed by mibefradil and NNC-55-0396 in a dose-dependent manner after 48 h treatment. However, mibefradil and NNC-55-0396 exhibited no effect on the growth of U937 and HEL cells which did not express T-type Ca^2+^ channels (Fig. 2c and d), suggesting that the anti-growth effect of both agents most likely resulted from blocking T-type Ca^2+^ channels of ALL cells.

**Fig. 3:**
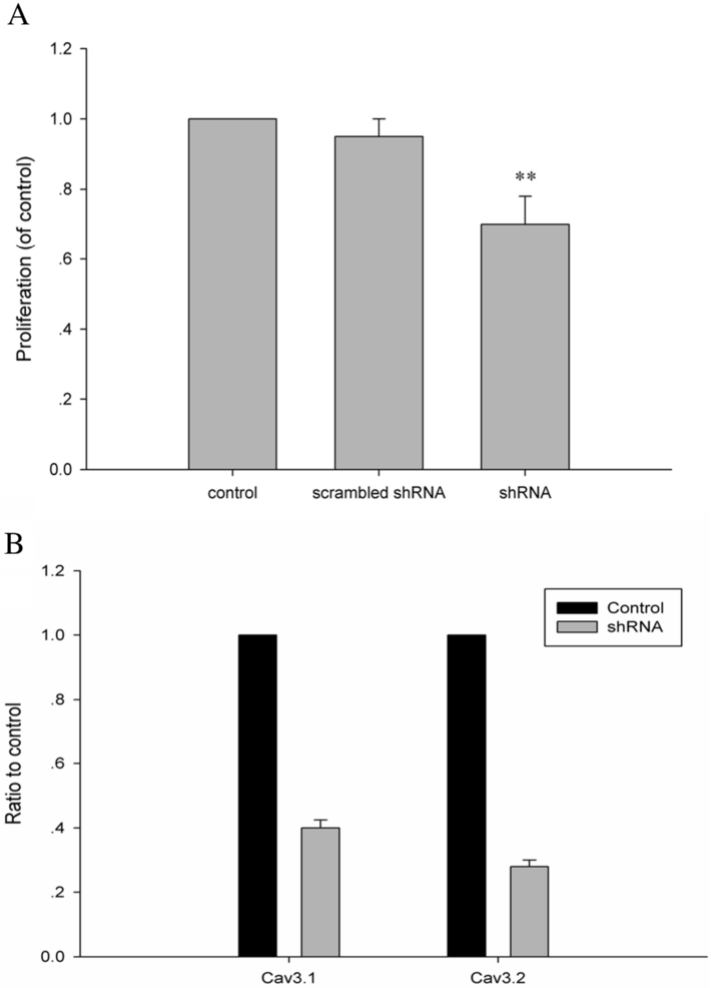
Effect of shRNA-induced Ca_v_3.1/Ca_v_3.2-gene silencing on MOLT-4 cell growth. **a** Transduction of shRNA into the MOLT-4 cells was achieved by a lentiviral infection method according to the manufacturer’s instructions. Cell growth was observed after 48 h growth in normal, shRNA-transduced, and scrambled-shRNA infection control group. **b** Q-PCR analysis of the level of Ca_v_3.1/Ca_v_3.2 knockdown. Data are mean ± SEM of three independent experiments in triplicates. **P < 0.01 versus scrambled-shRNA control group

To further demonstrate that T-type Ca^2+^ channels are indeed involved in ALL cell growth, we treated MOLT-4 cells with shRNA targeting to both a1G and a1H (a1G/H) to knocked down T-type Ca^2+^ channels. As shown in Fig. 3a, shRNA-transduced cells had significantly lower growth rates compared to the scrambled-shRNA infection and vehicle control group. Together, these results suggest that the functional T-type Ca^2+^ channels contribute to the growth of human T cell leukemia lines.

### Mibefradil and NNC-55-0396 inhibited ALL cell growth via cell cycle arrest and inducing cell apoptosis

**Fig. 4:**
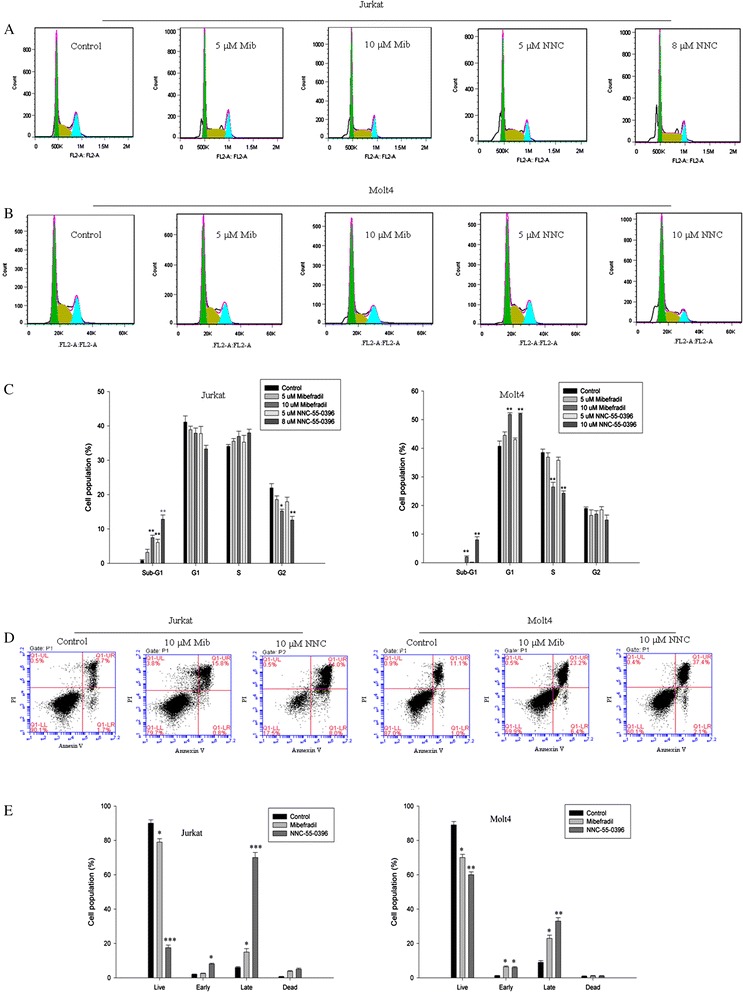
Effect of T-type Ca^2+^ channel antagonists, mibefradil (Mib) and NNC-55-0396 (NNC), on the cell cycle distribution and cell apoptosis of Jurkat and MOLT-4 cells. **a-c** Each cell was treated with different concentrations (0, 5 or 10 μM) of mibefradil or NNC-55-0396 for 48 h. To acquire enough cells for cell cycle analysis, Jurkat cells were treated with 8 μM NNC-55-0396. Cell cycle phase was determined using flow cytometry (FACS). **d-e** Cells were stained with Annexin V -FITC and propidium iodide (PI) after treatment with 10 μM mibefradil or NNC-55-0396 for 48 h (except Jurkat cells subjected to NNC-55-0396 for 24 h). Flow cytometry profiles represent annexin V-FITC staining in x-axis and PI in y-axis for the three experimental conditions. Percentage of each LR and UR area represents Annexin V-positive/PI-negative (early apoptotic) and Annexin V-positive/PI-positive cells (late apoptotic), respectively. Mibefradil and NNC-55-0396 had a dual effect on cell viability: (a) reducing the proliferation rate; and (b) increasing cell apoptosis. **c and e** Histogram bars representing the mean ± SEM of three independent experiments. *P < 0.05, **P < 0.01, ***P < 0.001 versus control group. Statistical significances were determined using one factor ANOVA and Student-Newman-Keuls post-test

To address how cell growth was inhibited by mibefradil and NNC-55-0396, cell cycle was examined. Mibefradil and NNC-55-0396 not only reduced the proliferation rate, but also induced apoptosis. After incubation with mibefradil or NNC-55-0396 for 48 h, the percentage of MOLT-4 cells in the G0/G1 phase was significantly enhanced, whereas that in the S phase was markedly reduced (Fig. 4b and c, right panel). Furthermore, both of the T-type Ca^2+^ channel antagonists induced a remarkable increase in the number of cells at the sub-G1 phase, a hallmark of cell apoptosis. While in Jurkat cells, mibefradil and NNC-55-0396 mainly induced cell apoptosis confirmed by a significant increase in the percentage of cells at the sub-G1 phase (Fig. 4a and c, left panel). The discrepancy of both antagonists on cell cycle of MOLT-4 and Jurkat cells may arise from the different expression level of T-type Ca^2+^ channels. The cytotoxicity of both inhibitors was also verified by FACS analysis of Annexin V-FITC and PI stained cells (Fig. 4d and e). In addition, cell death following treatment with both inhibitors was also confirmed by characteristics of apoptosis, such as cell shrinkage and chromatic agglutination (data not shown). Together these results indicate that mibefradil and NNC-55-0396 had a dual role on cell viability: (a) blunting proliferation; and (b) promoting cell apoptosis.

### Mibefradil and NNC-55-0396 down-regulated ERK signaling pathway in MOLT-4 cells

**Fig. 5:**
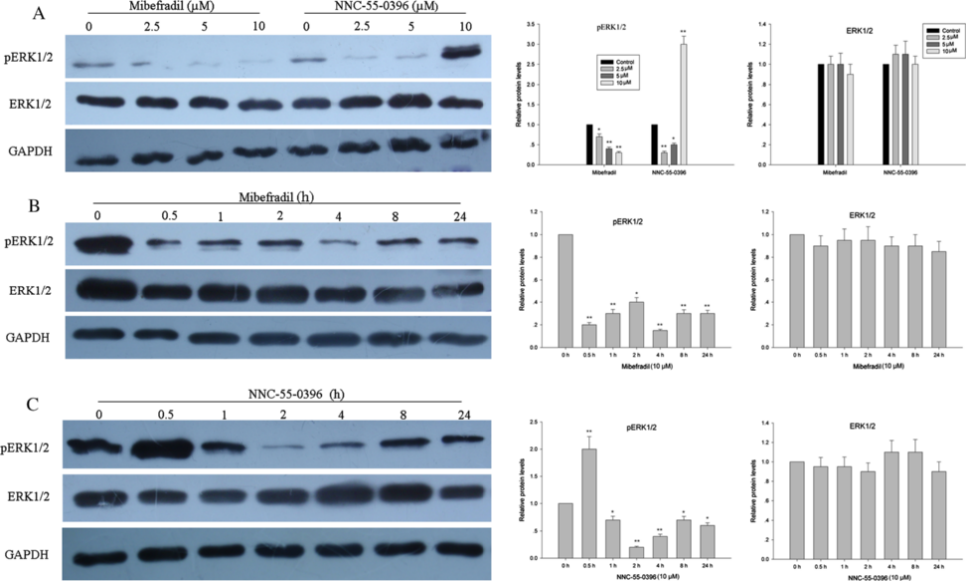
T-type Ca^2+^ channel antagonists, mibefradil and NNC-55-0396 modulated phospho-p44/42 MAP kinase activation in MOLT-4 cells. **a** MOLT-4 cells were incubated with different concentration of mibefradil or NNC-55-0396 for 48 h. **b-c** MOLT-4 cells were treated with 10 μΜ Mibefradil or NNC-55-0396 for various time-points from 0 to 24 h. Cell lysates were separated by SDS-PAGE and transferred to PVDF membranes. Membranes were probed with a phosphospecific Ab to detect activated Erk1/2 (top panel). The membrane was stripped and reprobed with an Ab directed against Erk1/2 to detect the total amount of kinase loaded in each lane (middle panel). GAPDH was used as a loading control for each lane (bottom panel). The results are presented as mean ± SEM of three independent experiments. *P < 0.05, **P < 0.01 compared with untreated control cells

It has been reported that Ca^2+^ can interact with the MAP kinase signaling pathway in T lymphocytes [28–30], and MAP kinase signaling pathway plays an important role in regulating cell cycle progression. Therefore we investigated whether T-type Ca^2+^ channel antagonists, mibefradil and NNC-55-0396 could modulate the expression of the p44/42 MAP kinase in MOLT-4 cells. As shown in Fig. 5b and c, a persistent decrease of phosphorylated ERK1/2 was detected after treatment with either inhibitor, except for a transient enhanced phosphorylation of ERK1/2 after NNC-55-0396 treatment. Furthermore, we found that mibefradil and NNC-55-0396 induced the decrease of pERK1/2 in a concentration-dependent manner except 10 μM NNC-55-0396, which induced a robust phosphorylation of ERK1/2 (Fig. 5a), in consistent with the Ca^2+^ overload after 10 μM NNC-55-0396 treatment (Additional file 3: Figure S3). These data suggest that mibefradil and NNC-55-03963 could modulate phosphor-p44/42 MAP kinase activation via regulating intracellular Ca^2+^ level, which may contribute to both inhibitor effects on cell growth in ALL cells.

### NNC-55-0396 induced endoplasmic reticulum calcium release

**Fig. 6:**
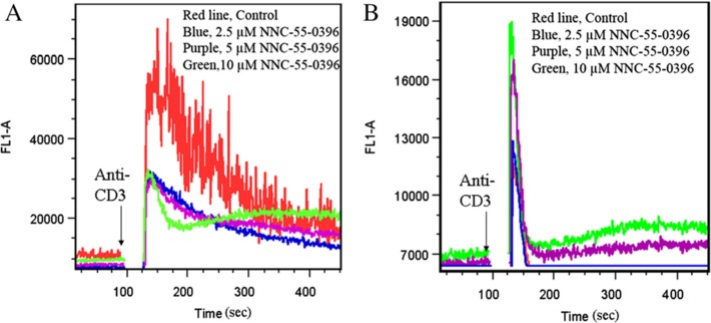
Effect of T-type Ca^2+^ channel antagonist, NNC-55-0396 on intracellular Ca^2+^ levels in Jurkat T cells. **a** Jurkat cells stained with Fluo-4 were preincubated with 2.5-10 μM NNC-55-0396 in the presence of extracellular Ca^2+^. For each sample, after the 10 min treatment with different concentrations of NNC-55-0396 baseline Ca^2+^ measurements were taken, cells were then stimulated at the 2 min mark with 10 μg/ml soluble anti-CD3 monoclonal antibody (mAb), OKT3 (R&D Systems, Minneapolis, MN, USA) to activate Ca^2+^ influx, and the analysis was immediately resumed. **b** Fluo-4 loaded Jurkat cells were treated with 2.5-10 μM NNC-55-0396 and stimulated in the absence of extracellular Ca^2+^. Results are representative of 3 independent experiments

Disruption of intracellular Ca^2+^ homeostasis is one of the primary processes in the early development of cell injury [31–33], and NNC-55-0396 had stronger cytotoxicity than mibefradil, especially for Jurkat cells. Thus, we examined the effect of NNC-55-0396 on intracellular Ca^2+^ level in Jurkat cells using flow cytometry. After NNC-55-0396 treatment, a dose-dependent increase in cytosolic Ca^2+^ concentration was seen in the absence of extracelluar Ca^2+^ (Fig. 6b). Moreover, low concentration NNC-55-0396 (<5 μM) decreased intracellular baseline Ca^2+^ levels, while high concentration NNC-55-0396 (>5 μM) diminished or abolished the inhibiting effect of intracellular baseline Ca^2+^ levels in the present of extracelluar Ca^2+^ (Fig. 6a). In addition, 10 μM NNC-55-0396 induced sustained Ca^2+^ overload (Fig. 6a, Green line). In fact, high concentration mibefradil and NNC-55-0396 also induced intracellular Ca^2+^ overload in MOLT-4 cells (Additional file 3: Figure S3).

**Fig. 7:**
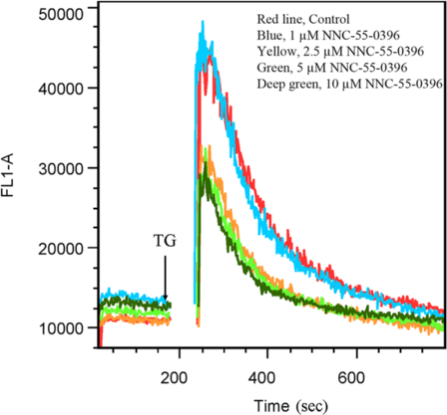
NNC-55-0396 exposure increased intracellular baseline Ca^2+^ levels via inducing ER calcium release. Jurkat T cells stained with Fluo-4 were preincubated with 1–10 μM NNC-55-0396 in the absence of extracellular Ca^2+^. For each sample, after the 10 min pretreatment with different concentrations of NNC-55-0396 baseline Ca^2+^ measurements were taken, cells were then stimulated at the 2 min mark with 1 μM thapsigargin (TG) to induce the endoplasmic reticulum (ER) Ca^2+^ release, and the analysis was immediately resumed. The results depicted are representative of three independent experiments

Because endoplasmic reticulum (ER) is the major Ca^2+^ store of intracellular Ca^2+^, and T-type Ca^2+^ channels have been suggested to couple Ca^2+^ influx to ER Ca^2+^ storage [34], we asked whether NNC-55-0396 would cause a disruption of Ca^2+^ homeostasis at the ER, ultimately leading to enhanced apoptosis. We first measured whether the response of Jurkat cells to thapsigargin (TG, an agent irreversibly inhibits (sarco) endoplasmic reticulum Ca^2+^-ATPase (SERCA) and depletes ER Ca^2+^ stores) could be altered by NNC-55-0396. As shown in Fig. 7, TG-driven increase in [Ca2+] i was attenuated when cells were pretreated with NNC-55-0396. Similar results were obtained with MOLT-4 cells (data not shown). These findings suggest that NNC-55-0396 depletes Ca^2+^ from the ER.

**Fig. 8:**
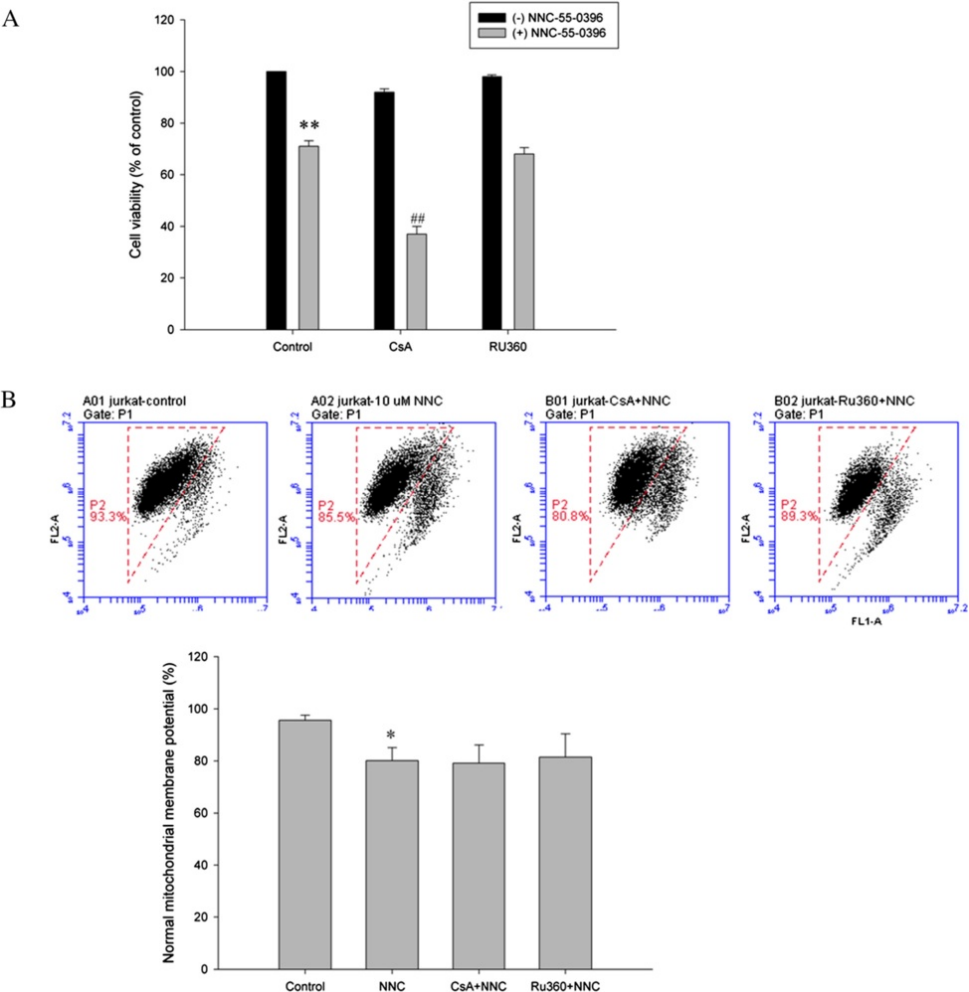
Effects of the mitochondrial uniporter antagonist RU360 and mitochondrial permeability transition pore (mPTP) inhibitor CsA on NNC-55-0396-induced cell apoptosis and depolarization of the mitochondrial membrane potential in Jurkat cells. **a** Live cells were examined by 2 parameters: forward scatter/side scatter (FSC/SSC) index of live cells in cell size and granularity by FACScan. Cells were preincubated with mitochondrial calcium uptake (Ru360, 20 μM) or mitochondrial permeability transition pore (mPTP) inhibitor (cyclosporine A [CsA], 1 μM) for 1 h, then incubated for 6 h in the presence of 10 μM NNC-55-0396. **b** Cells were preincubated with mitochondrial calcium uptake (Ru360, 20 μM) or mitochondrial permeability transition pore (mPTP) inhibitor (CsA, 1 μM) for 1 h, then incubated for 2 h in the presence of 10 μM NNC-55-0396. Then the mitochondrial membrane potential was determined by FACS. Results are presented as mean ± SEM of four independent experiments. **p < 0.01 versus control group (−) NNC-55-0396, ^##^ p < 0.01 versus control group (+) NNC-55-0396, *p < 0.05 versus control group

A proximal target of Ca^2+^ signals arising from the ER is the mitochondrial network [35]. Several observations underline the significance of the role of this ER-mitochondrial Ca^2+^ flux in stimulating apoptosis [36]. Therefore we decided to address whether NNC-55-0396-induced ER Ca^2+^ release had a putative effect on depolarization of the mitochondrial membrane potential, resulting in cell apoptosis. To this end, we first measured the effect of NNC-55-0396 on mitochondrial membrane potential in Jurkat cells. As shown in Fig. 8b, NNC-55-0396 induced depolarization of the mitochondrial membrane potential significantly compared to control after incubation for 2 h (P < 0.05).

To test whether NNC-55-0396 induced depolarization of the mitochondrial membrane potential arises from ER Ca^2+^ release, which disrupts mitochondrial Ca^2+^ homeostasis, Ru360 (20 μM, mitochondrial calcium uptake inhibitor) or cyclosporine A (CsA, 1 μM, mitochondrial permeability transition pore (mPTP) inhibitor) was preincubated with Jurkat cells for 1 h before NNC-55-0396 treatment. Unexpectedly, neither of the compounds had significantly protective effect on mitochondrial membrane potential (Fig. 8b). In addition, neither of the compounds had any protective effect on cell viability (Fig. 8a). Inversely, CsA enhanced the cytotoxicity of NNC-55-0396 in Jurkat cells, may attribute to the inhibition effect of CsA on Calcineurin. In addition, neither of the compounds had any protective effect on mitochondrial membrane potential and cell viability in MOLT-4 cells (Additional file 4: Figure S4). These findings demonstrate that the effect of NNC-55-0396 on depolarization of the mitochondrial membrane potential may not directly depend on ER Ca^2+^ release.

## Discussion

In the present investigation, we have identified the expression of T-type Ca^2+^ channels in human leukemic cell lines. We also demonstrated that T-type Ca^2+^ channel antagonists, mibefradil and NNC-55-0396 not only reduced the proliferation of ALL cells, but also induced apoptosis. Furthermore, mibefradil and NNC-55-0396 disrupted intracellular calcium homeostasis, partially from ER Ca^2+^ release. Mibefradil and NNC-55-0396 modulated phospho-p44/42 MAP kinase activation in MOLT-4 T cells. Our study provides a potential that T-type Ca^2+^ channels may be a potential target for ALL therapy.

Cancer cells have been reported to be relatively insensitive to reductions in extracellular calcium concentration [37]. Ca^2+^-dependent signalling is frequently deregulated in cancer cells and, importantly, voltage-gated calcium channels (VGCCs) may play a role in remodelling Ca^2+^ homeostasis. Abnormal up-regulation of the gene encoding T-type Ca^2+^ channel was detected in various tumour cells [38], suggesting that T-type Ca^2+^ channels play a role in cancer development.

In the present study, because MOLT-4 cells expressed high level of T-type Ca^2+^ channels, they were used for patch-clamp recording analysis. The patch-clamp recording results demonstrate that the current in MOLT-4 cells activated at −30 mV, with peak current at 0 mV, inconsistent with other reports of recording T-current [11, 13, 39]. The discrepancy may have arisen from different cell lines used in the study. Furthermore, current-clamp recordings show that the mean resting potential was −30.5 ± 1.8 mV in MOLT-4 cells. In addition, the flow cytometric calcium flux assay indicates that cultured T-ALL cells displayed a basal Ca^2+^ influx which can be reduced by T-type Ca^2+^ channel blockers. Together, these results are consistent with the occurrence of T-type Ca^2+^ channel window currents, providing the pattern of Ca^2+^ signaling required for cell cycle progression.

Several studies with in vitro systems have demonstrated that antagonists of T-type Ca^2+^ channels reduce cancer cell proliferation and viability [40]. In addition, inhibition of T-type Ca^2+^ channels with mibefradil had been shown to induce apoptosis in breast cancer cells [41] and glioblastoma cells [10]. This observation supports the idea that T-type Ca^2+^ channels function as regulators of survival and/or apoptosis signaling. In this study, blocking the functional T-type Ca^2+^ channels significantly decreased the growth of Jurkat and MOLT-4 cells, while mibefradil and NNC-55-0396 had no effect on the growth in U937 and HEL cells, which didn’t express T-type Ca^2+^ channels. These results demonstrate a strong correlation between T- type Ca^2+^ channels expression and growth inhibition. Interestingly, we found that the lower-expression cell line (Jurkat) showed a larger growth inhibition than the higher-expressing cell line (MOLT-4), especially for NNC-55-0396 treatment. The phenomenon may attribute to NNC-55-0396-induced Ca^2+^ release in Jurkat cells, resulting in a larger cell death. In addition, the high percentage of sub-G1 phase upon NNC-55-0396-treatment also indicates that the Jurkat cell death is due to its inherent strong cytotoxicity as well as T-type Ca^2+^ channel blockade. Cell cycle analysis data demonstrated that mibefradil and NNC-55-0396 had a dual effect on cell viability: (a) decreasing proliferation rate; (b) inducing cell apoptosis. As shown in Fig. 4b and c (right panel), mibefradil and NNC-55-0396 inhibited MOLT-4 cells proliferation rate through a halt in the progression to the G1-S phase.

Ca^2+^ is an essential regulator of the cell cycle and is indispensable for cell proliferation. For example, the transition from the G1/S interphase (initiation of DNA synthesis) and the G2/M interphase (initiation of mitosis), is dependent upon Ca^2+^/calmodulin-dependent kinase II (CaM-kinase II) [42]. In proliferating cells, these Ca^2+^ signals are often organized in oscillatory patterns involving entry of external Ca^2+^ and release of Ca^2+^ from internal stores. T-type Ca^2+^ channels are particular well suited to participate in such oscillations due to their low voltage activation ranges, transient kinetics of inactivation and “window current”. Indeed, many proliferating cells exhibit T-type Ca^2+^ current, including a variety of tumour cells [38, 40]. As shown in Additional file 2: Figure S2 and Fig. 6a, blocking T-type Ca^2+^ channels with pharmacological blockers reduced intracellular calcium concentration, confirming the role of these channels in calcium concentration maintenance.

Mibefradil was originally presented as a T-type Ca^2+^ channel blocker and has been used in many studies to establish this putative causal link between T-type Ca^2+^ channels and cell proliferation. However, mibefradil has also been reported to inhibit cell proliferation through an association with cell swelling and the inhibition of volume-sensitive Cl^−^ channels [43, 44] or several other ion channels [45–47]. Son *et al*. reported that NNC-55-0396 inhibited voltage-dependent K^+^ channels in rabbit coronary arterial smooth muscle cells [48]. Thus, the inhibitory effects on cell proliferation of non-specific T-type Ca^2+^ channel blockers should be carefully attributed to T-type Ca^2+^ channel blockage.

In general, the alterations of Ca^2+^ homeostasis have long been associated with apoptotic cell death [49]. For example, a larger and more prolonged Ca^2+^ changes (Ca^2+^ surge or Ca^2+^ overload) could trigger cell death. Therefore, the question arises, why blocking T-type Ca^2+^ calcium channels, which should inhibit calcium influx from the external environment, paradoxically induces an extensive apoptotic response in ALL cells? One possible explanation lies on the fact that cytosolic Ca^2+^ can be increased not only through influx from outside, but also via release of calcium ions from the internal stores. As shown in Fig. 6b and Fig. 7, NNC-55-0396 could increase cytosolic Ca^2+^ level from inducing ER Ca^2+^ release. In addition, mibefradil at high concentration (≥10 μM) also induced intracellular Ca^2+^ overload (Additional file 3: Figure S3). These results are consistent with a recent report that mibefradil at supratherapeutic concentrations (≥10 μM) induced Ca^2+^ release from IP3R-operated Ca^2+^ stores in rat cardiac fibroblasts and human platelets *in vitro* [50]. Furthermore, the work by Das *et al.* in melanoma cells demonstrated that mibefradil and pimozide both induce ER stress followed by autophagy, culminating in apoptotic cell death [51]. Valerie *et al.* reported that targeting T-type Ca^2+^ channels inhibits mTORC2/Akt pro-survival signaling pathways and induces apoptosis [10]. It appears that both the specificity of the inhibitor and the properties of the model system used may determine the final cellular response to T-type Ca^2+^ channel blockage: cell cycle arrest, apoptosis, autophagy, necrosis, or any combination of them.

The ER and mitochondria are crucial nodes at which intracellular Ca^2+^ fluxes are governed and are the principal locations for signaling cell fate choices. In addition, a proximal target of Ca^2+^ signals arising from the ER is the mitochondrial network. Thus the potential involvement of mitochondria was also determined. It is known that exposure of mitochondria to high Ca^2+^ concentrations results in their swelling and uncoupling. This phenomenon leads to a loss of maintenance of cellular ATP levels and finally to cell death by necrosis [52]. In our study, Ru360, a specific mitochondrial calcium uptake inhibitor (uniport transporter inhibitor) and cyclosporine A (mPTP inhibitor) were not associated with any effect on NNC-55-0396 toxicity, suggesting that mitochondrial calcium uptake may not be involved in the toxicity in our model. In addition, ER stress, as a result of chronic depletion of Ca^2+^ from the ER, is also a signal for cell death. The work by Das *et al.* showed that T-type channel inhibition or down-regulation results in the activation of the IRE1 pathway (giving rise to XBP-1 s) and, possibly, also of the protein kinase RNA-like ER kinase (PERK) or ATF6 pathways of the UPR (inducing GADD153) [51]. Thus ER stress may play an important role in inducing cell apoptosis in our study. Because Ca^2+^ has close association with MAPK signaling pathway, we next investigated whether mibefradil and NNC-55-0396 can modulate MAP kinase activity. MAP kinase signaling pathway plays an important role in regulating cell cycle progression, and T-type Ca^2+^ channel inhibitors blunted cell proliferation—through a halt in the progression to the G1-S phase in MOLT-4 cells, so MOLT-4 cells were used as a model to study ERK signaling pathway. We report here that both inhibitors down-regulated ERK signaling pathway in MOLT-4 cells, in agreement with Kotturi report that inhibition of Ca^2+^ influx decreased the phosphorylation of ERK1/2 [28]. Since ERK1/2 plays an important role in regulating cell proliferation, the inhibition of ERK1/2 signaling pathway may be associated with the proliferation inhibition of MOLT-4 cells with mibefradil and NNC-55-0396 treatment.

## Conclusions

We have shown both molecular and extensive pharmacological evidence for the presence of a T-type Ca^2+^ channel in leukemia cell lines. Mibefradil and NNC-55-0396 had a dual role on cell viability: (a) inhibiting cell proliferation; (b) promoting cell apoptosis. Mechanistically, both T-type Ca^2+^ channel inhibitors induced ER Ca^2+^ release and disrupted ERK1/2 signaling pathway. Based on these observations and *in vivo* results reported elsewhere, we propose that T-type Ca^2+^ channel blockers may be utilized as future therapies for neoplasm expressing T-type channels.

## Additional files

Additional file 1:
**Electrophysiological recordings from MOLT-4 T cells.** (A) Traces showing typical recording of the T-type Ca^2+^ current (Ba^2+^ current) triggered from a holding potential of −80 mV to 30 ms-long depolarizing steps at −60 to +30 mV (10 mV increments) with an interpulse interval of 2 s in 20 mM Ba^2+^-containing bathing solution. (B) A plot of the current–voltage relationship for the Ca^2+^ current recorded as detailed in (A). Additional file 2:
**Effect of T-type Ca** ^**2+**^ **channel antagonist, mibefradil on** **intracellular Ca** ^**2+**^ **levels in Jurkat T cells.** Jurkat T cells stained with Fluo-4 were preincubated with 0.5-10 μM mibefradil in the presence of extracellular Ca^2+^. For each sample, after the 10 min treatment with different concentrations of mibefradil baseline Ca^2+^ measurements were taken, cells were then stimulated at the 2 min mark with 10 μg/ml soluble anti-CD3 monoclonal antibody (mAb), OKT3 to activate Ca^2+^ influx, and the analysis was immediately resumed. Results are representative of 3 independent experiments. Additional file 3:
**Effect of T-type Ca** ^**2+**^ **channel antagonists, mibefradil and NNC-55-0396** **on intracellular Ca** ^**2+**^ **levels in MOLT-4 T cells.** Graphs show the effect of high concentration mibefradil and NNC-55-0396 on the intracellular baseline Ca^2+^ levels in the presence of extracellular Ca^2+^. Results are representative of 3 independent experiments. Additional file 4:
**Effects of the mitochondrial uniporter antagonist RU360 and mitochondrial permeability transition pore (mPTP) inhibitor CsA on NNC-55-0396-induced cell apoptosis and depolarization of the mitochondrial membrane potential in MOLT-4 cells.** (A) Live cells were examined by 2 parameters: forward scatter/side scatter (FSC/SSC) index of live cells in cell size and granularity by FACScan. Cells were preincubated with mitochondrial calcium uptake (Ru360, 20 μM) or mitochondrial permeability transition pore (mPTP) inhibitor (cyclosporine A [CsA], 1 μM) for 1 h, then incubated for 12 h in the presence of 10 μM NNC-55-0396. (B) Cells were preincubated with mitochondrial calcium uptake (Ru360, 20 μM) or mitochondrial permeability transition pore (mPTP) inhibitor (CsA, 1 μM) for 1 h, then incubated for 8 h in the presence of 10 μM NNC-55-0396. Then the mitochondrial membrane potential was determined by FACS. Results are presented as mean ± SEM of four independent experiments. **p < 0.01 versus control group (−) NNC-55-0396, *p < 0.05 versus control group.

## Abbreviations

ALL: Acute lymphocytic leukemia
ER: Endoplasmic reticulum
PBMC: Peripheral blood mononuclear cell
PI: Propidium iodide
PERK: RNA-like ER kinase
UPR: Unfolded protein response
TG: Thapsigargin
CsA: Cyclosporine A
VGCC: Voltage-gated calcium channel
