# Supplementary figures and images for: T-type calcium channel antagonists, mibefradil and NNC-55-0396 inhibit cell proliferation and induce cell apoptosis in leukemia cell lines
Source: J Exp Clin Cancer Res. 2015 May 21;34(1):54. doi: 10.1186/s13046-015-0171-4 (PMC4443536; doi:10.1186/s13046-015-0171-4)

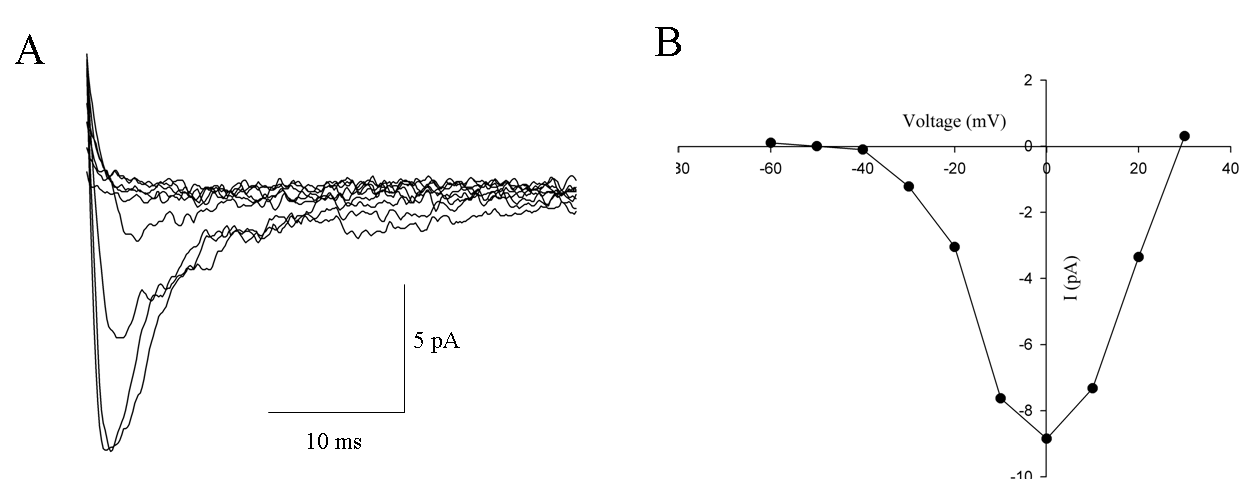

Supplement: Additional file 1: — Electrophysiological recordings from MOLT-4 T cells. (A) Traces showing typical recording of the T-type Ca2+ current (Ba2+ current) triggered from a holding potential of −80 mV to 30 ms-long depolarizing steps at −60 to +30 mV (10 mV increments) with an interpulse interval of 2 s in 20 mM Ba2+-containing bathing solution. (B) A plot of the current–voltage relationship for the Ca2+ current recorded as detailed in (A). [file 13046_2015_171_MOESM1_ESM.tif]

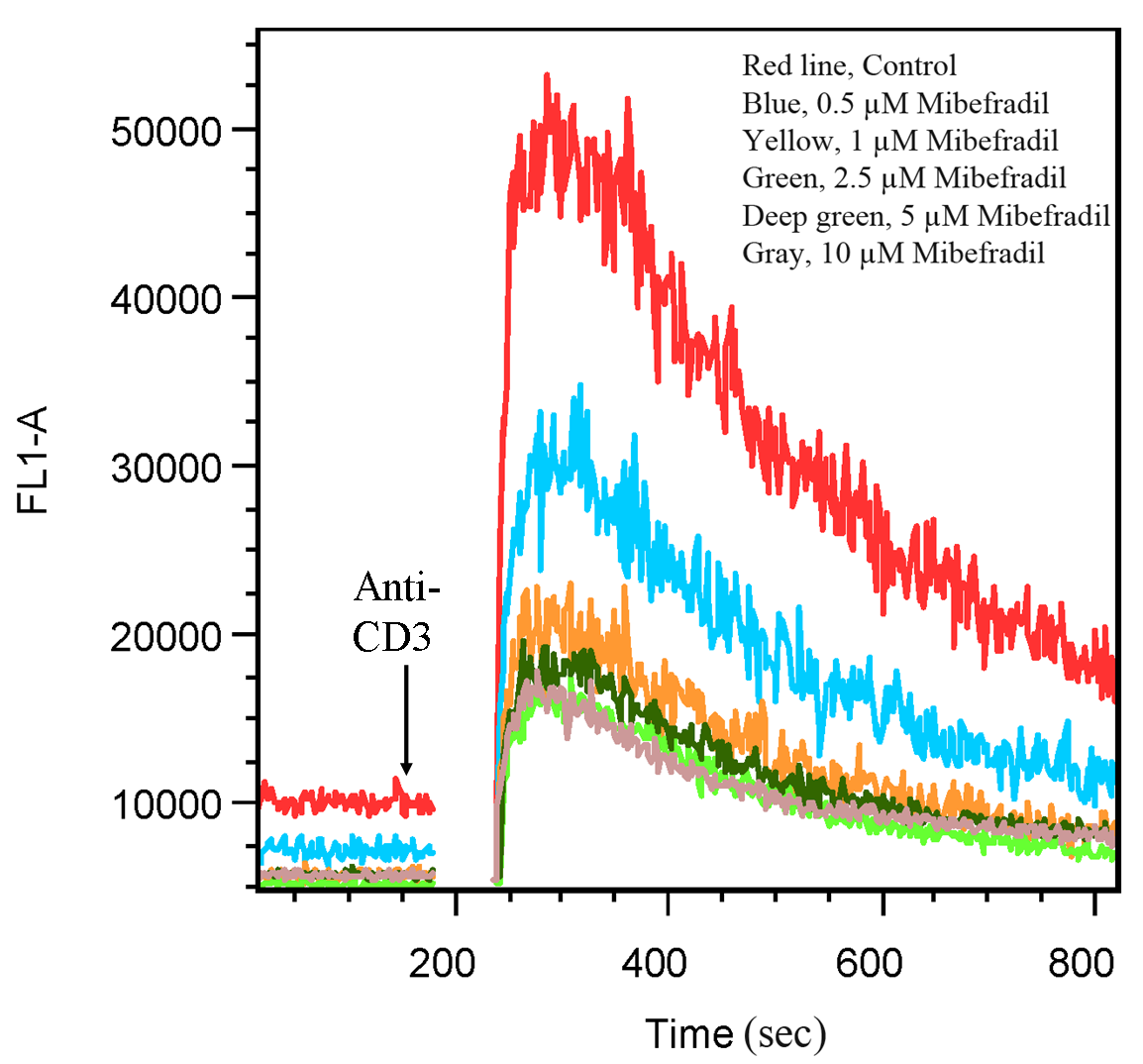

Supplement: Additional file 2: — Effect of T-type Ca 2+ channel antagonist, mibefradil on intracellular Ca 2+ levels in Jurkat T cells. Jurkat T cells stained with Fluo-4 were preincubated with 0.5-10 μM mibefradil in the presence of extracellular Ca2+. For each sample, after the 10 min treatment with different concentrations of mibefradil baseline Ca2+ measurements were taken, cells were then stimulated at the 2 min mark with 10 μg/ml soluble anti-CD3 monoclonal antibody (mAb), OKT3 to activate Ca2+ influx, and the analysis was immediately resumed. Results are representative of 3 independent experiments. [file 13046_2015_171_MOESM2_ESM.tif]

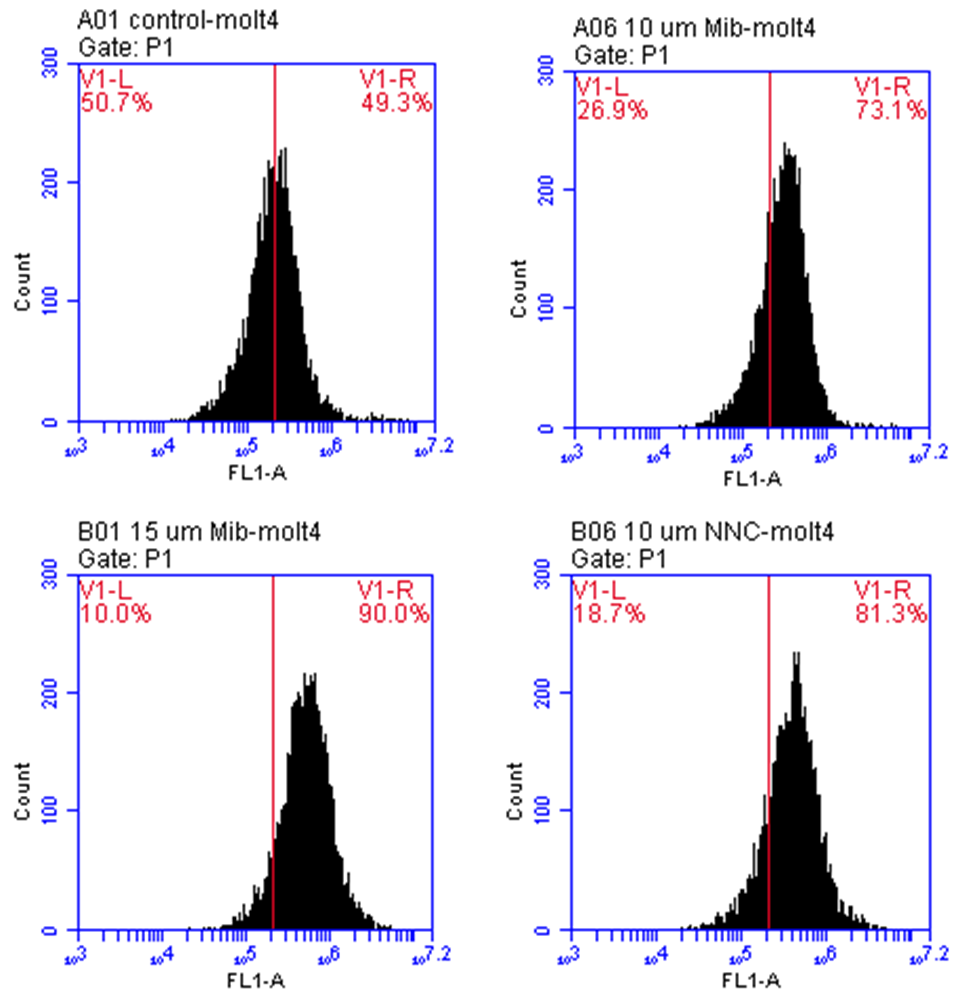

Supplement: Additional file 3: — Effect of T-type Ca 2+ channel antagonists, mibefradil and NNC-55-0396 on intracellular Ca 2+ levels in MOLT-4 T cells. Graphs show the effect of high concentration mibefradil and NNC-55-0396 on the intracellular baseline Ca2+ levels in the presence of extracellular Ca2+. Results are representative of 3 independent experiments. [file 13046_2015_171_MOESM3_ESM.tif]

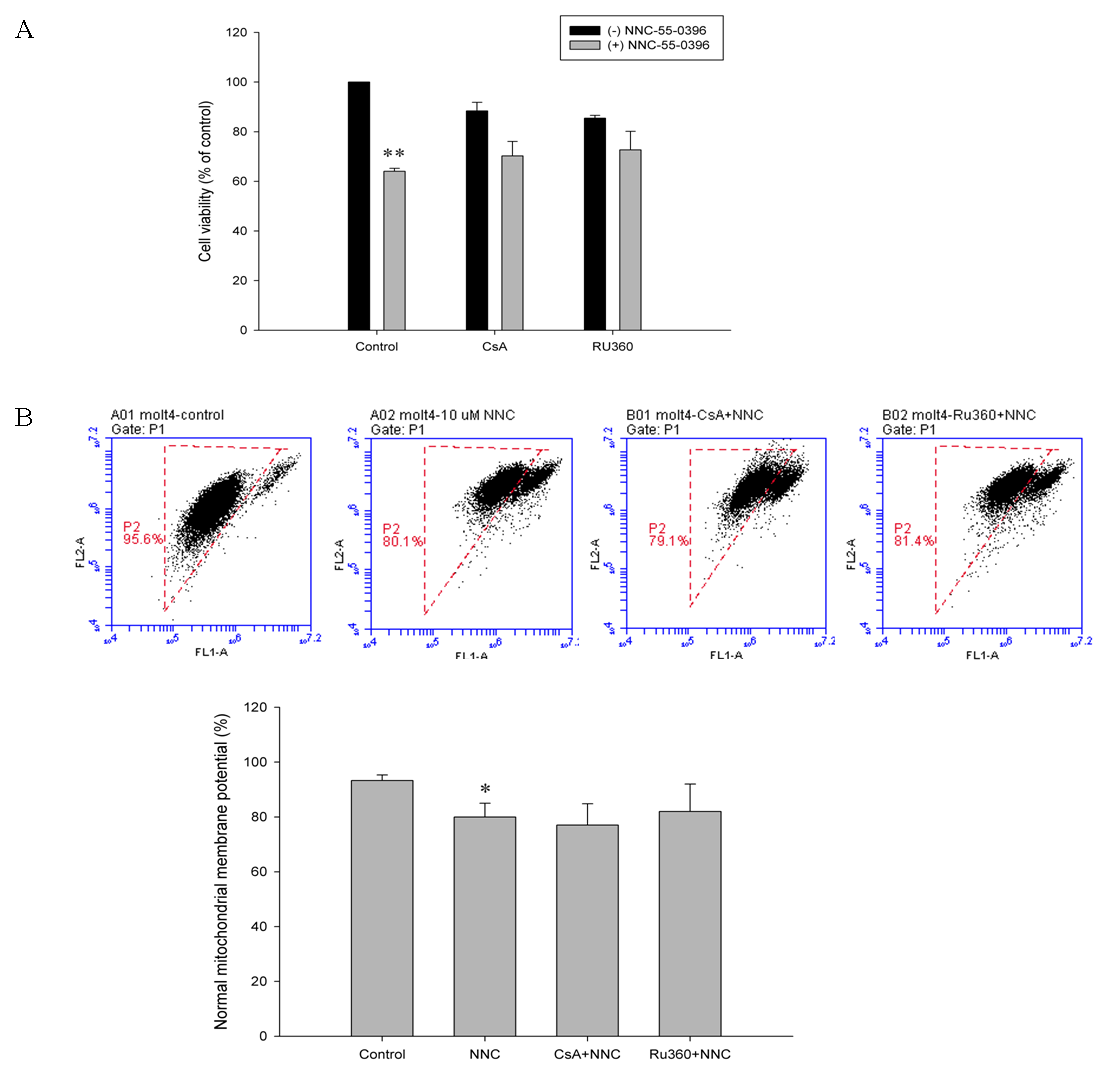

Supplement: Additional file 4: — Effects of the mitochondrial uniporter antagonist RU360 and mitochondrial permeability transition pore (mPTP) inhibitor CsA on NNC-55-0396-induced cell apoptosis and depolarization of the mitochondrial membrane potential in MOLT-4 cells. (A) Live cells were examined by 2 parameters: forward scatter/side scatter (FSC/SSC) index of live cells in cell size and granularity by FACScan. Cells were preincubated with mitochondrial calcium uptake (Ru360, 20 μM) or mitochondrial permeability transition pore (mPTP) inhibitor (cyclosporine A [CsA], 1 μM) for 1 h, then incubated for 12 h in the presence of 10 μM NNC-55-0396. (B) Cells were preincubated with mitochondrial calcium uptake (Ru360, 20 μM) or mitochondrial permeability transition pore (mPTP) inhibitor (CsA, 1 μM) for 1 h, then incubated for 8 h in the presence of 10 μM NNC-55-0396. Then the mitochondrial membrane potential was determined by FACS. Results are presented as mean ± SEM of four independent experiments. **p < 0.01 versus control group (−) NNC-55-0396, *p < 0.05 versus control group. [file 13046_2015_171_MOESM4_ESM.tif]
